# Transcriptomic profile analysis of the left atrium in spontaneously hypertensive rats in the early stage

**DOI:** 10.3389/fphar.2022.989636

**Published:** 2022-10-17

**Authors:** Qinghua Fang, Jing Wang, Jiangjun Wei, Xianglin Long, Yao Wang, Jiacheng He, Xin Yuan, Jianlin Du

**Affiliations:** ^1^ Department of Cardiology, The Second Affiliated Hospital of Chongqing Medical University, Chongqing, China; ^2^ Department of Nephrology, The Second Affiliated Hospital of Chongqing Medical University, Chongqing, China

**Keywords:** hypertension, left atrium, transcriptome, atrial fibrosis, cardiac metabolism changes

## Abstract

Left atrial remodeling, characterized by enlargement and hypertrophy of the left atrium and increased fibrosis, was accompanied by an increased incidence of atrial fibrillation. While before morphological changes at the early stage of hypertension, how overloaded hypertension influences the transcriptomic profile of the left atrium remains unclear. Therefore, RNA-sequencing was performed to define the RNA expressing profiles of left atrium in spontaneously hypertensive rats (SHRs) and normotensive Wistar-Kyoto (WKY) rats as a control group. We also compared the changes in the RNA expression profiles in SHRs treated with an angiotensin receptor blocker (ARB) and angiotensin receptor-neprilysin inhibitor (ARNI) to assess the distinct effects on the left atrium. In total, 1,558 differentially expressed genes were found in the left atrium between WKY rats and SHRs. Bioinformatics analysis showed that these mRNAs could regulate upstream pathways in atrial remodeling through atrial fibrosis, inflammation, electrical remodeling, and cardiac metabolism. The regulated transcripts detected in the left atrial tissue in both the ARB-treated and ARNI-treated groups were related to metabolism. In contrast to the ARB-treated rates, the transcripts in ARNI-treated rats were mapped to the cyclic guanosine monophosphate-protein kinase G signaling pathway.

## 1 Introduction

Hypertension is the most important controllable risk factor in cardiovascular disease (Mills et al., 2020). Persistent blood pressure overload in hypertensive patients may induce left ventricle hypertrophy, heart failure, enlargement of the left atrium, arrhythmia (especially atrial fibrillation, AF), and cardiovascular death (Kamioka et al., 2018; Parker et al., 2020; Kario and Williams, 2021). Hypertension can rapidly induce atrial remodeling, including left atrial hypertrophy, fibrosis, and an inflammatory response (Gumprecht et al., 2019; Kim et al., 2019; Wu et al., 2021). Short-term and moderate stress overload pressure results in ultrastructural changes in left atrial cells before structural remodeling of the left ventricle (Aguas et al., 1981). Hypertension is the most significant population-attributable risk factor for AF that is independent and potentially controllable (Rahman et al., 2016). However, the mechanism that allows hypertension to lead to AF remains unclear. Therefore, identifying the transcriptional characteristics of the left atrium in the early stage of hypertension may help to reveal the atrial arrhythmia substrate induced by overloaded pressure.

Single-cell RNA-seq and bulk RNA-seq were used to delineate the transcriptomic profiles of heart and aorta in hypertensive animal models, systematically revealing the mechanisms of cardiac vascular remodeling, including activation of fibroblasts and vascular smooth muscle cells, dysregulation of interactions between macrophages and T cells, which were linked to multiple signaling pathways, such as TGF-β signaling pathway, cytokine, MAPK Signaling pathway (Costa Ade and Franco, 2015; Li et al., 2016; Xu et al., 2018; Cheng et al., 2021). Heart failure model, the most typical cardiac remodeling model, revealed multiple mechanisms involved in cardiac remodeling by transcriptome sequencing, including activation of myofibroblast (Chothani et al., 2019; Ramanujam et al., 2021) and immune cells (Martini et al., 2019; Abplanalp et al., 2021), mitochondrial dysfunction (Sweet et al., 2018; Zhuang et al., 2022), proinflammatory signaling (Costa Ade and Franco, 2015; Hahn et al., 2021) and TGF-β signaling pathway (Stratton et al., 2019). Although there have been many studies on RNA-seq in exploring the mechanism in target organ remodeling in hypertension, the transcriptomic characteristics of hypertension-induced atrial remodeling are still lacking.

Given the close link between hypertension and AF, antihypertensive drugs may potentially reduce the risk of AF, especially the renin–angiotensin–aldosterone system inhibitor because of its anti-myocardial remodeling effect (Rahman et al., 2016; Seccia et al., 2017). Both angiotensin II type 1 receptor antagonists and sacubitril/valsartan were demonstrated to attenuate adverse cardiac remodeling by reversing cardiac fibroblasts and hypertrophy (Kusaka et al., 2015; Garvin et al., 2021). Sacubitril/valsartan was proven to be superior in reducing left ventricular hypertrophy because it targets both the renin–angiotensin system and neprilysin, and thus this therapy has an advantageous cardiovascular prognosis in patients with hypertension compared with unitary treatment using olmesartan (Schmieder et al., 2017). However, the specific mechanisms associated with reverse cardiac remodeling under angiotensin receptor blocker (ARB) or sacubitril/valsartan treatment remain unclear.

**FIGURE 1:**
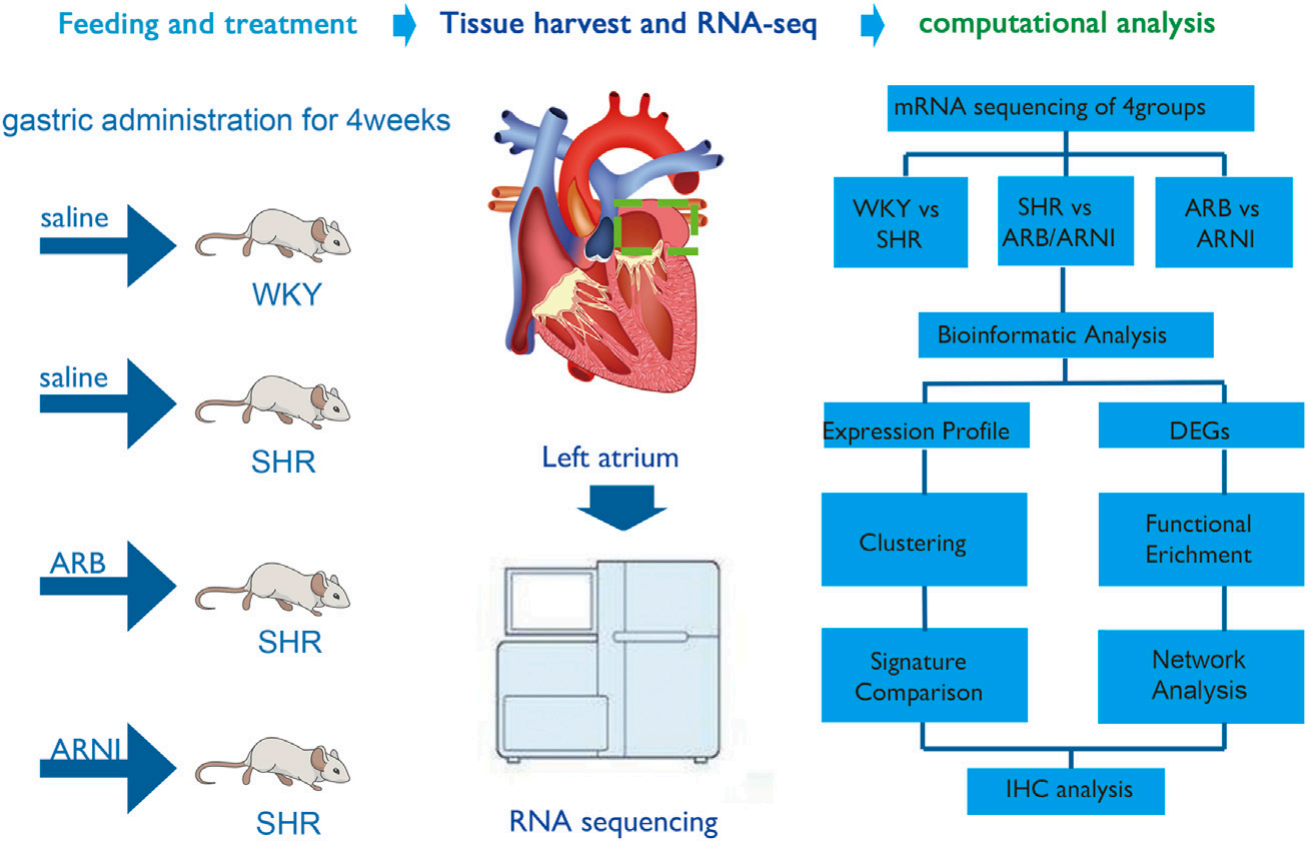
An overall flow chart of the experimental design. WKY, Wistar-Kyoto; SHR, spontaneously hypertensive rat; ARB, angiotensin receptor blocker; ARNI, angiotensin receptor/neprilysin inhibitor; DEGs, differentially expressed genes; IHC, immunohistochemistry.

In the present study, we conducted RNA-seq to compare the transcriptional differences in the left atrium in spontaneously hypertensive rats (SHRs) and Wistar-Kyoto (WKY) rats (Figure 1). Furthermore, we characterized the biological functions of these differentially expressed genes (DEGs) by bioinformatics analysis to further understand the effects of hypertension on the left atrium. In addition, transcriptome analysis was performed for the left atrium tissues of SHRs fed saline, ARB, and sacubitril/valsartan. Bioinformatics analysis was also performed to demonstrate the changes in gene expression associated with the different treatments in order to elucidate the mechanisms responsible for atrial remodeling under treatment with ARB and sacubitril/valsartan. Furthermore, we compared the transcriptional differences in rats under different treatments to identify their distinct effects on the left atrium of ARB and sacubitril/valsartan. This transcriptomic profile of left atrium enables a more furtherly understand its mechanism of development of left atrial transcriptional remodeling in early hypertension.

## 2 Materials and methods

### 2.1 Experimental animals

Fourteen-week-old male SHRs (N = 9) and WKY rats (N = 3) were purchased from Vital River Laboratory Animal Technology Co. Ltd. (Beijing, China). The first group comprising the normotensive control (WKY, N = 3), was fed with saline (7.5 ml/kg/day) routinely and independently for 4 weeks. The SHRs were randomly divided into three groups: which were fed with saline (7.5 ml/kg/day, N = 3), valsartan (30 mg/kg/day, N = 3) and sacubitril/valsartan (60 mg/kg/day, N = 3) for 4 weeks. All animal protocols were approved by the Animal Research Ethics Committee of Chongqing Medical University.

### 2.2 Histological analysis

Immediately after anesthetizing the rats by intraperitoneal injection of 20% ethyl carbamate, the left atrium was ablated through thoracotomy and a portion was immersed in ice-cold isolation buffer, which was rapidly frozen at −80°C to prepare for RNA-seq. Other parts of the atrial tissue were fixed in 8% neutral formaldehyde and embedded in paraffin. After dewaxing, the paraffin sections were stained with hematoxylin and eosin (H&E) and Masson’s trichrome. The sections were observed under a microscope at 200× lens (Zhu et al., 2018).

H&E staining was conducted for histological determination of myocardial injury by quantifying the ratio of the inflammatory cell infiltration and necrosis area relative to the entire field as described in previous studies (Rezkalla et al., 1988), as follows: score 0 = 0 (no myocardial damage observed); score 1 = 0%–25%; score 2 = 25%–50%; score 3 = 50%–75%; score 4 = 75%–100% (Supplementary Table S1). Similarly, the extent of myocardial fibrosis was quantified by Media Cybernetics (United States) using the Masson’s trichrome. The ratio of the collagen fiber area was calculated as the area with positive staining for collagen relative to the entire visual field of the section (Takemoto et al., 1997). The area density was defined as the integral optical density (IOD) divided by the pixel area. One section was randomly selected from each rat in the four groups. Three different fields in each section were selected for scoring according to the criteria above.

### 2.3 RNA sequencing

Total RNA was extracted from the left atrium using TRIzol (Invitrogen, Carlsbad, CA, United States) according to the manufacturer’s instructions, and then quality controlled and quantified using a NanoDrop and Agilent 2100 bioanalyzer (Thermo Fisher Scientific, MA, United States), respectively. RNA-seq was conducted by a commercially available service (service ID: F21FTSCCWLJ1374_MOUmpqzN, BGI-Shenzhen, China). Briefly, after breaking the total RNA into short fragments, mRNA was enriched using oligo (dT) magnetic beads, followed by cDNA synthesis. Double-stranded cDNA was purified and enriched by PCR amplification, after which the library products were sequenced using a BGIseq-500. The sequencing data were filtered with SOAPnuke (v1.5.2). The clean reads were mapped to the reference genome using HISAT2 (v2.0.4). Bowtie2 (v2.2.5) was applied to align the clean reads to the reference coding gene set, and the expression levels of genes were then calculated using RSEM (v1.2.12). All RNA-seq data has been uploaded to the GEO database and can be queried through GSE207283.

### 2.4 Data analysis

The raw counts were used to calculate the expression level of each gene, and DESeq2 (v1.4.5) was employed to compare the expression levels of genes between different samples. DEGs were filtered using the following criteria: log2FC ≥ 1 and Q value ≤ 0.05. The DAVID online analysis tool was used to perform functional cluster analysis for the DEGs between the WKY and SHR groups. The biological functions of DEGs were determined according to the significantly enriched Gene Ontology (GO) terms (http://www.geneontology.org/). Fisher’s exact and multiple comparison tests were used to calculate the significance level (*p*-value) and false positive rate (FDR) for each function, and the significant functions of DEGs were screened using the threshold of *p* < 0.05. Pathway analysis was conducted based on the Kyoto Encyclopedia of Genes and Genomes (KEGG; http://www.genome.jp/kegg/) to explore the significant pathways. Pathways with FDR ≤ 0.5 were defined as significantly enriched. Gene set enrichment analysis (GSEA) was performed using software (Subramanian et al., 2005) to quantify the normalized enrichment score and FDR. Principal component analysis, volcano plot and protein–protein interaction network analysis were performed in BGI online system (Dr.Tom).

Key driver gene analysis (KDA) was carried out using BGI online system (Dr.Tom). Specifically, KDA analysis takes as input a set of genes (G) and a directed gene network (N), aiming at identifying the key regulators of the gene set associated with a given network (Rual et al., 2005; Tran et al., 2011). The size of h-layer neighborhood (HLN) for each node was calculated. The value of HLN is equal to the number of downstream nodes in the range h away from the specific nodes. The nodes are selected as candidate drivers if their HLN values are greater than $\overset{-}{\mu }$ + *σ* (*μ*), where μ is defined as the composite set of HLNS of all nodes, $\overset{-}{\mu }$ is the mean value of *μ*, and *σ*(*μ*) is the standard deviation of μ. The candidate drivers without any root node are global drivers, which is defined as key driver genes, while the rest were local drivers. Nodes with out-degree above $\overset{-}{d}$ +2*σ* (d) are global driver genes, where d is defined as the set of out-degrees of all nodes, $\overset{-}{d}$ is the mean of d, and *σ* (d) as the standard deviation of d.

### 2.5 Immunohistochemistry analysis

Immunohistochemistry techniques were used to study the expression of transforming growth factor-β (TGF-β). Specimens were incubated overnight with primary antibodies at 4°C and then incubated with horseradish peroxidase-labeled secondary antibodies at room temperature for 1 h. DAB color developing solution was used for the chromogenic reaction. The antibodies comprised the primary antibody anti-TGF-β1 rabbit antiserum (Servicebio, China) and secondary antibody horseradish peroxidase-conjugated goat anti-rabbit immunoglobulin G (Servicebio, China). Sections were then processed by microscopy (Nikon) and analyzed with Aipathwell digital pathology image analysis software. The mean density was defined as the integrated optical density divided by the quantity of positive cells. Six different fields were selected for quantitative analysis in each group.

## 3 Result

### 3.1 Expression profiling

**FIGURE 2:**
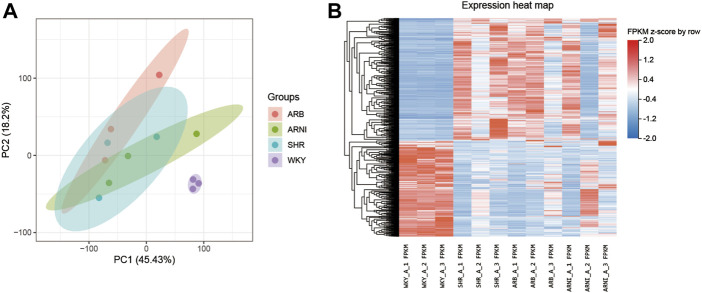
An overview of the transcriptomic landscape profile in the rats’ left atrium. **(A)** PCA analyse of transcriptome profiles between the four groups: the abscissa and ordinate represent different principal components and their contribution proportions. **(B)** Heatmap indicating the atlas of dysregulated genes in the four groups: the abscissa represents different groups of rats, the ordinate represents different genes, red represents up-regulated genes, and blue represents down-regulated genes. PCA, principal component analysis; WKY, Wistar-Kyoto; SHR, spontaneously hypertensive rat; ARB, angiotensin receptor blocker; ARNI, angiotensin receptor/neprilysin inhibitor; FPKM, fragments per kilobase of exon per million reads mapped.

PCA showed a closer distance on the scatter plot among groups than between groups, thereby indicating that there were significant differences between the atrial tissues from normotensive and spontaneous hypertension rats (Figure 2A). The transcriptional differences in the four groups are shown in Figure 2B and Supplementary Figure S1.

### 3.2 Morphology of the left atrium in different groups

**FIGURE 3:**
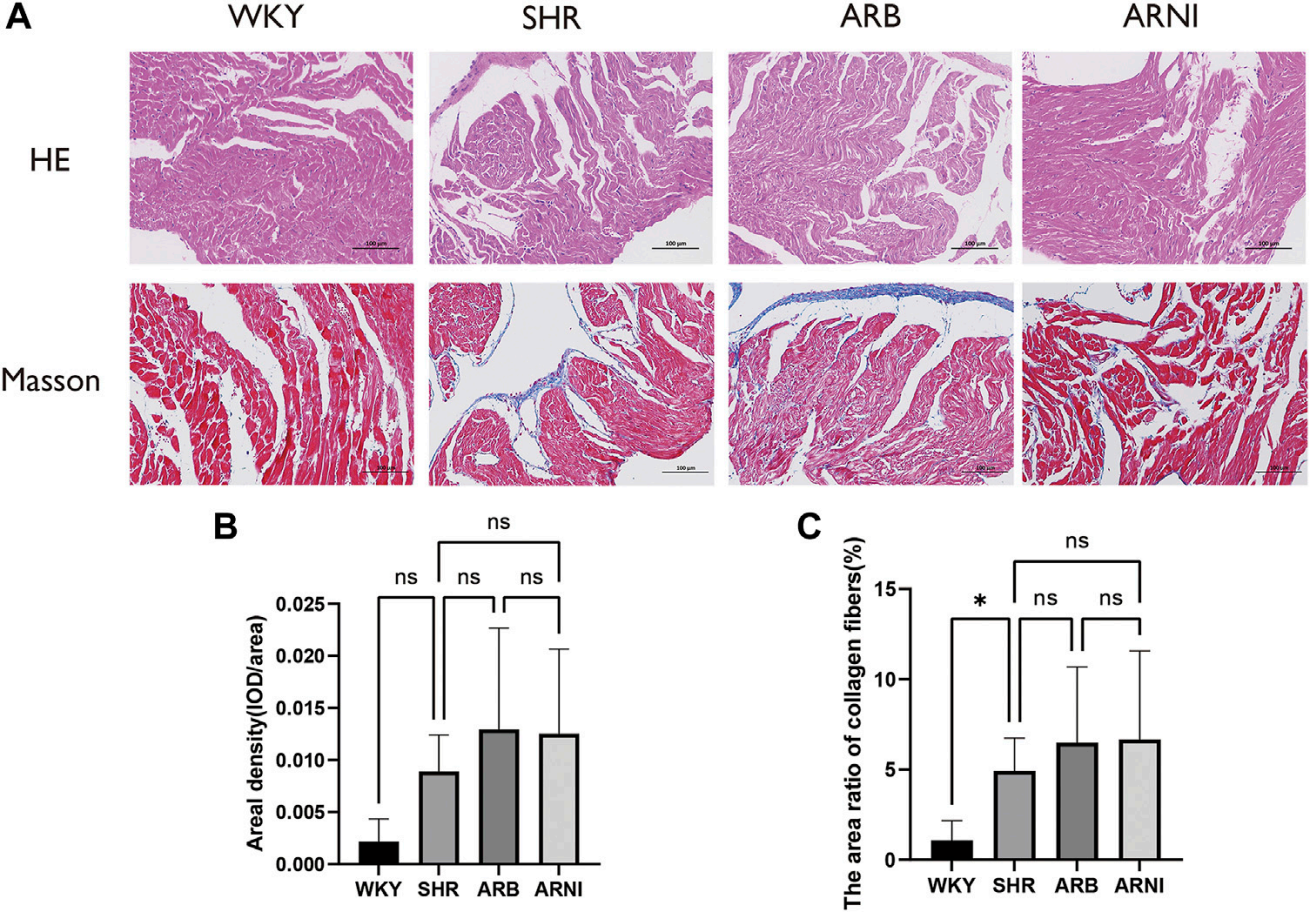
Morphology of the left atrium in different groups. **(A)** The left atriums were harvested for H&E and Masson’s trichrome. **(B)** Quantitative analyses of areal density (IOD/area) in Masson’s trichrome in four groups. **(C)** Quantitative analyses of the area ratio of collagen fibers in Masson’s trichrome in four groups. H&E, hematoxylin-eosin; WKY, Wistar-Kyoto; SHR, spontaneously hypertensive rat; ARB, angiotensin receptor blocker; ARNI, angiotensin receptor/neprilysin inhibitor; IOD, integral optical density; ns, no significance. *: *p* ≤ 0.05.

H&E was used to evaluate histological cardiac damage and inflammation (Supplementary Table S2). The results of H and E results indicated no apparent necrosis or inflammatory infiltration in the left atrial tissue under light microscopy in any group. Further quantitative analysis detected no significant differences among the four groups (Figure 3A). Masson’s trichrome was used to estimate the degree of myocardial fibrosis (Supplementary Tables S3, S4). The mean area density values did not differ significantly in the SHR, ARB-treated, and ARNI-treated groups compared with the WKY group (Figure 3B). The area ratio of collagen fibers was slightly elevated in SHRs compared with WKY rats (Figure 3C). These results suggest that the hypertension overloading pressure did not lead to significant histological changes manifested as inflammation and fibrosis in the left atrium in the early development stage of hypertension development. Early administration of inhibitors of the renin–angiotensin system did not influence the histology of the left atrium.

### 3.3 Distinct transcriptomic changes in the atrial tissues of SHRs

**FIGURE 4:**
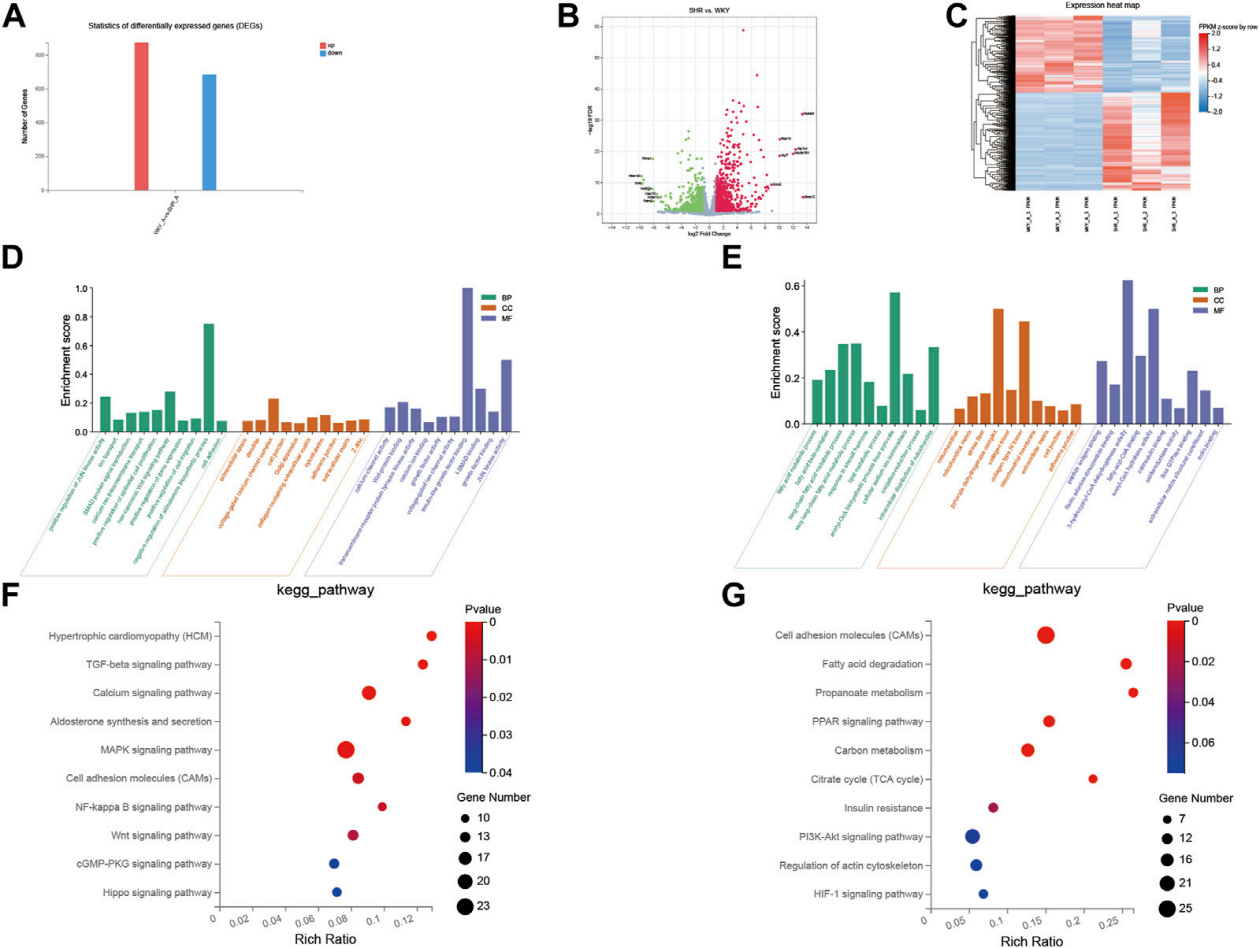
GO and KEGG pathway analysis of DEGs between SHR and WKY rat left atrium. **(A)** Histogram of the numbers of DEGs: the abscissa represents the direction of gene change, the ordinate represents the number of genes, red represents up-regulation, blue represents down-regulation; **(B)** volcano Plot: *X*-axis represents the fold change of log2, *Y*-axis represents −log10 (*p*-value), red represents up-regulated genes, blue represents down-regulated genes, and gray represents insignificant genes; **(C)** heatmap: *X*-axis represents different groups of rats, *Y*-axis represents different genes, red represents up-regulated genes, blue represents down-regulated genes. **(D,E)** Ten GO enriched terms in the upregulated **(D)** and downregulated **(E)** DEGs, were analyzed for biological process (BP), cellular component (CC), and molecular function (MF). The *X*-axis shows different pathways, and the *Y*-axis shows enrichment scores, BP in green, CC in orange, and MF in blue. **(F,G)** 10 KEGG pathways were identified in the downregulated **(F)** and upregulated **(G)** DEGs. The *X*-axis represents the enrichment ratio, the *Y*-axis represents the different pathways enriched, the size of the dots represents the number of genes enriched to the pathway, and the change of color from blue to red represents the change of *p* value from small to large. GO, gene ontology; KEGG, Kyoto Encyclopedia of Genes and Genomes. TGF-beta, transforming growth factor beta; MAPK, mitogen-activated protein kinase; PPAR, peroxisome proliferator-activated receptor; TCA, tricarboxylic acid; HIF-1, hypoxia-inducible factor-1.

The gene expression profiles for the left atrium tissue were compared in the normotensive and spontaneously hypertensive groups to characterize the effects of hypertension overload pressure on the transcription levels (Figures 4A–C). In total, 1,558 DEGs were observed in the SHR group compared with the WKY group, where 873 genes were upregulated and 685 were downregulated.

GO assignments were used to classify the genes associated with the transformation of the left atrium in the SHRs. As shown in Figures 4D,E, compared with the WKY rats, the upregulated transcripts in SHRs were enriched in 1) biological process (BP): calcium ion transmembrane transport, noncanonical Wnt signaling pathway, cell adhesion and SMAD protein signal transduction; 2) molecular function (MF): calcium channel activity, Wnt-protein binding, voltage-gated ion channel activity, and I-SMAD binding; and 3) cellular component (CC): voltage-gated calcium channel complex, cell junction, and collagen-containing extracellular matrix. The downregulated transcripts were enriched in: 1) BP: fatty acid metabolic process, lipid metabolic process, oxidation–reduction process, and intracellular distribution of mitochondria; 2) MF: fatty-acyl-CoA binding, calmodulin binding, and extracellular matrix structural constituent; and 3) CC: mitochondrion, extracellular matrix, cell junction, and mitochondrial membrane.

We performed pathway enrichment analysis with KEGG to further characterize the DEGs. As shown in Figures 4F,G, in the left atrial tissues of these hypertensive rats, compared with the WKY group, the upregulated transcripts were related to the TGF-β signaling pathway, calcium signaling pathway, MAPK signaling pathway, and NF-kappa B signaling pathway, whereas the downregulated transcript were related to the PPAR signaling pathway, fatty acid metabolism, carbon metabolism, PI3K-Akt signaling pathway, and apelin signaling pathway.

### 3.4 Network analysis of DEGs and enriched pathways between the WKY and SHR groups

**FIGURE 5:**
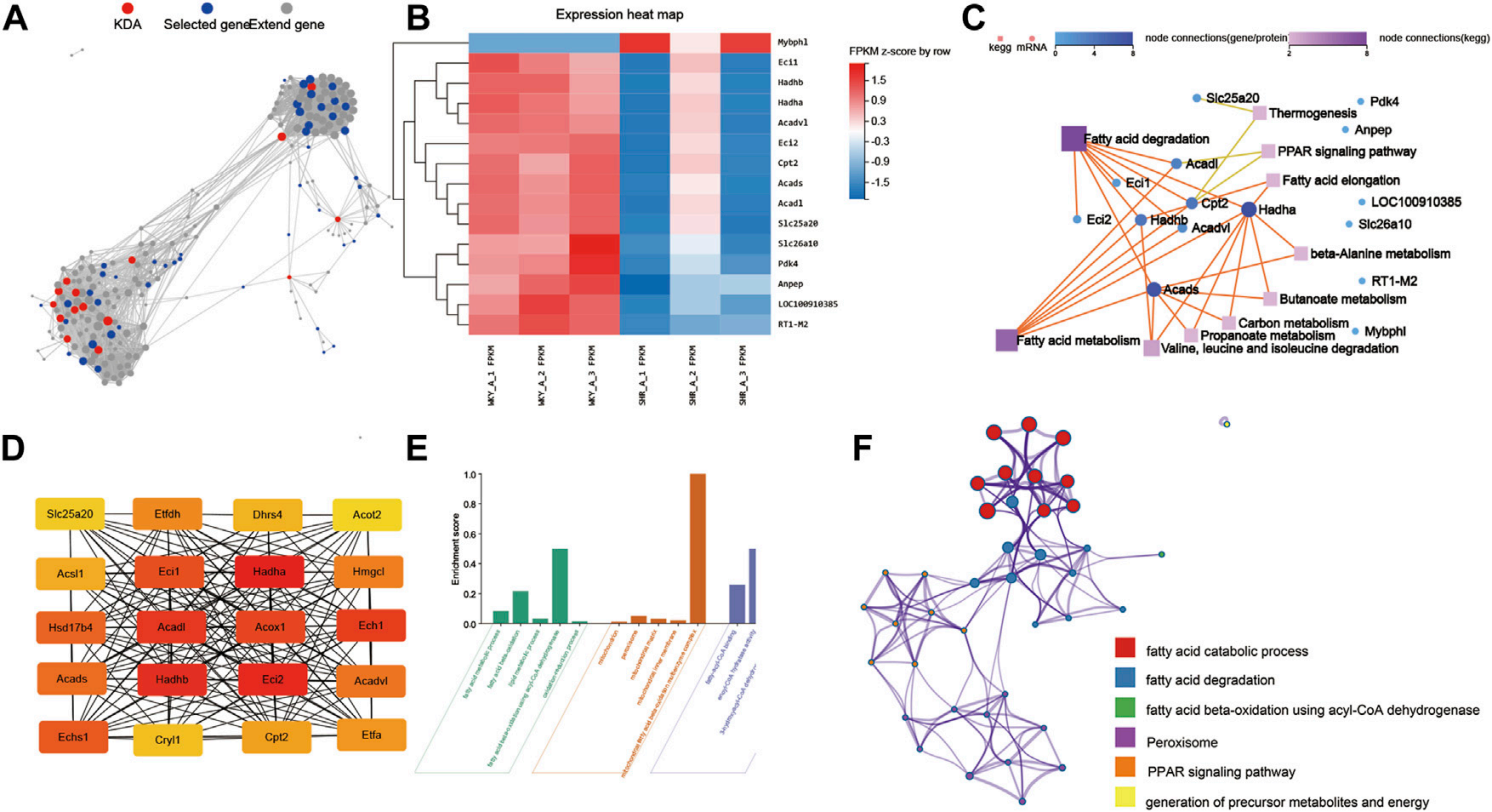
Network analysis of DEGs and enriched pathways between THE WKY and SHR groups. **(A)** KDA analysis identified 15 key driver genes in the DEGs between SHR and WKY rat atria: Key driver genes are shown in red, initial genes are shown in blue, and extended genes are shown in gray. **(B)** Heatmap of the 15 key driver genes. **(C)** Network analysis between key driver genes and enriched KEGG pathways: Dots represent genes, squares represent KEGG pathways, and lines between genes and squares indicate that the gene can be enriched into the corresponding pathway. **(D)** The top 20 hub genes were selected by the cytoHubba app in Cytoscape 3.8.2 software. The node color changed gradually from yellow to red in ascending order according to the score of hub genes. **(E)** GO and enrichment network of the top 20 hub genes between SHR and WKY rat atrium. **(F)** The enrichment network of top 20 hub genes was identified in the rat atrium. Nodes represent functions enriched for an annotated ontology term and node size indicates the number of genes that fall into that term. KDA, key driver gene analysis; WKY, Wistar-Kyoto; SHR, spontaneously hypertensive rat; KEGG, kyoto encyclopedia of genes and genomes; BP, biological process; CC, cellular component; MF, molecular function; MAPK, mitogen-activated protein kinase.

Based on the protein–protein interaction network analysis of all the dysregulated genes (1,558 genes) in SHRs and the WKY rats, we selected 15 key driver genes in the prominent regulatory position (Figures 5A,B). We performed KEGG pathway analysis for further characterize the key genes. The interaction network obtained between the significantly enriched KEGG pathways and the genes determined by KDA is shown in Figure 5C. The key genes were enriched in pathways including fatty acid metabolism, and PPAR signaling pathway.

The CytoHubba app in Cytoscape 3.8.2 software was used to select the hub genes among the 1558 dysregulated genes in the left atrium in hypertensive rats. Twenty hub genes were selected, including *Hadha, Hadhb, Eci2*, and *Acadl* (Figure 5D). As shown in Figure 5E, GO analyses were conducted and they identified, GO-BP terms: fatty acid metabolic process, fatty acid beta-oxidation and lipid metabolic process; GO-MF terms: fatty-acyl-CoA binding and enoyl-CoA hydratase activity; GO-CC terms: mitochondrion, peroxisome and mitochondrial matrix. In addition, the top 20 GO and KEGG pathways of the 30 hub genes were identified by Metascape (Figure 5F) included the fatty acid catabolic process, fatty acid degradation and PPAR signaling pathway.

### 3.5 Sacubitril/valsartan and ARB modulate the transcriptomes in atrial tissue with spontaneous hypertension

**FIGURE 6:**
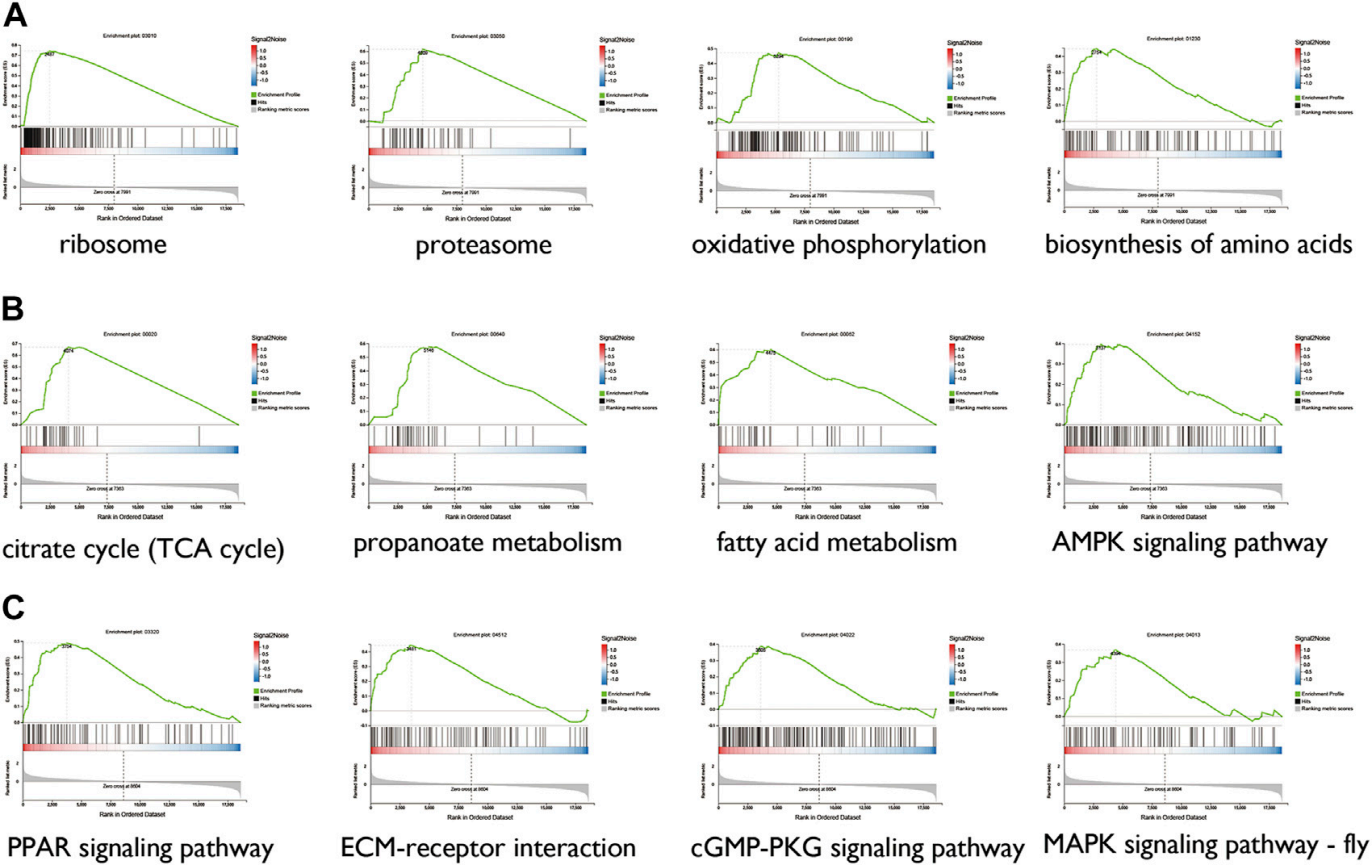
GSEA between the ARB-treated group and the SHR **(A)**, sacubitril/valsartan-treated group and the SHR **(B)**, and the ARB-treated and sacubitril/valsartan-treated groups **(C)** TCA, tricarboxylic acid; AMPK, AMP-activated protein kinase; PPAR, peroxisome proliferator-activated receptor; ECM, extracellular matrix; MAPK, mitogen-activated protein kinase.

Gene set enrichment analysis (GSEA) was performed to assess the concentration of genes regulated by sacubitril/valsartan and ARB in different gene sets in the KEGG pathways. As shown in Figure 6A, after the 4 weeks of treatment with ARB, the atrial tissues were mainly regulated in ribosome, proteasome, oxidative phosphorylation, and biosynthesis of amino acids. In the sacubitril/valsartan-treated group, the changes were mainly in the citrate cycle, AMPK signaling pathway, fatty acid elongation, propanoate metabolism, carbon metabolism, and PPAR signaling pathway (Figure 6B). We note that the pathways enriched in the ARB and ARNI groups were both involved in cardiometabolic pathways, thereby suggesting that the two drugs may contribute to repairing damage to the left atrium through this common mechanism.

Furthermore, GSEA was performed to compare the different mRNAs between the ARB- and sacubitril/valsartan-treated groups to identify the differences in atrial remodeling reversal mechanisms. As shown in Figure 6C, compared with ARB, mRNAs regulated in the sacubitril/valsartan-treated group were mainly enriched in the PPAR signaling pathway, ECM-receptor interaction, cGMP-PKG signaling pathway, and MAPK signaling pathway.

### 3.6 Immunohistochemistry analysis

**FIGURE 7:**
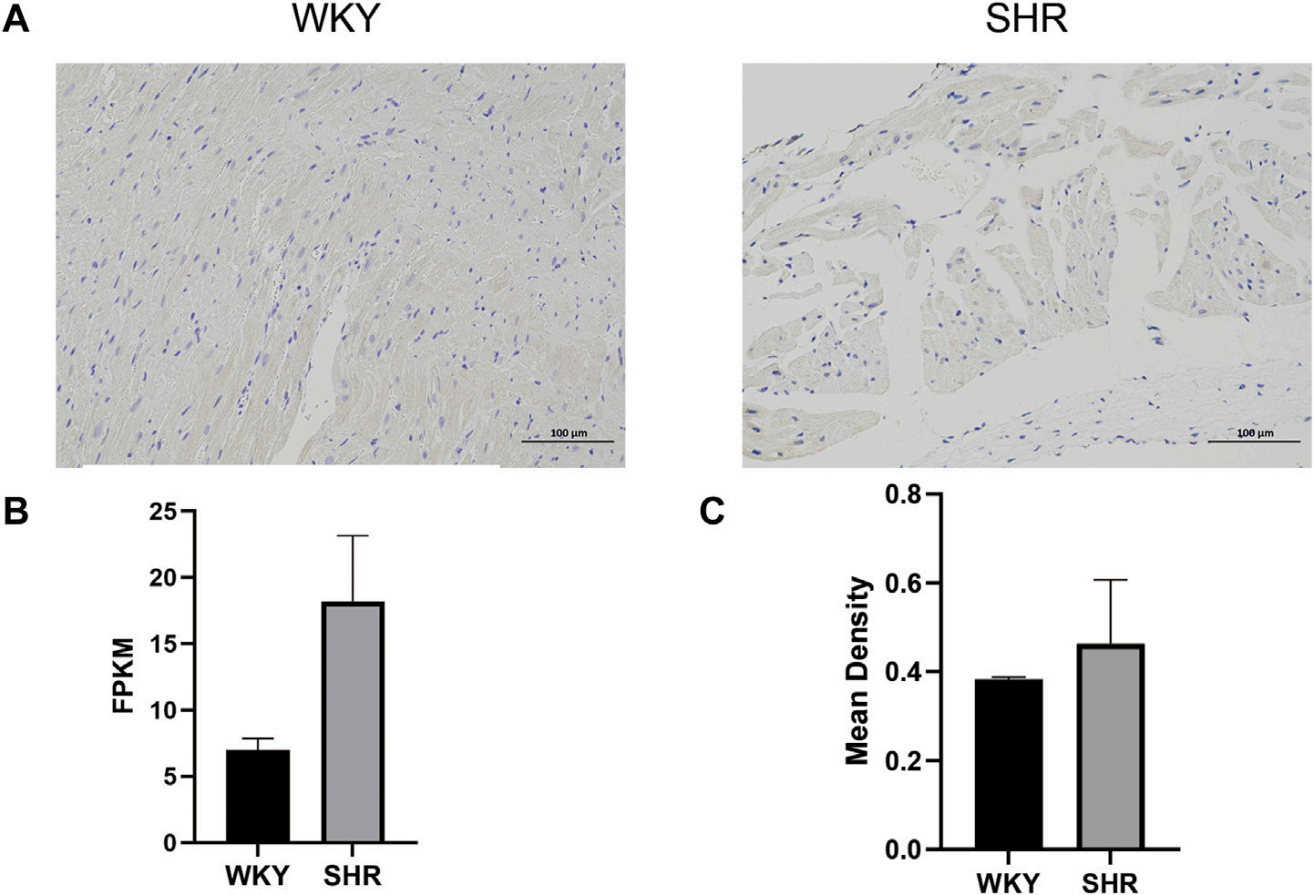
**(A)** Immunohistochemical analysis of the left atrium in different groups: the TGF-beta-positive cells manifested as buffy, and the nucleus was stained blue. **(B)** Expression levels of TGF beta in SHR and WKY: the *X*-axis represents different groups, and the *Y*-axis represents FPKM. **(C)** Mean density of Immunofluorescence staining: the *X*-axis represents different groups, and the *Y*-axis represents the mean density. WKY, Wistar-Kyoto; SHR, spontaneously hypertensive rat; FPKM, fragments per kilobase of exon per million reads mapped.

The TGF-β-positive cells manifested as brown and the nucleus was stained blue by immunohistochemical staining (Supplementary Table S5). As shown in Figure 7, the expression level of TGF-β was higher in the left atrium of SHRs compared with normotensive rats, which was consistent with the changes in the gene expression levels.

## 4 Discussion

### 4.1 Influence of hypertension on the left atrium

Complex changes in the atrium increase the susceptibility and progression to AF, and stimulate AF-associated diseases, and thus they are defined as “atrial cardiomyopathy” according to a recent consensus study (January et al., 2019). The multidirectional association between elevated blood pressure and AF has not been elucidated, and the main theories currently focus on complex associations such as structural remodeling, electrophysiology, neuroendocrine, inflammation, and autonomic mechanisms (Dzeshka et al., 2017). Transcriptome and proteome analyses were used to comprehensively understand the changes caused by hypertension and to further study the AF substrate in hypertension (Alvarez-Franco et al., 2021). In a previous study, Julio et al. observed 15 altered proteins in the early stage of left ventricular hypertrophy in SHRs compared with normotensive rats by proteomic analysis, and they mediated hypertension-induced cardiac hypertrophy (Gallego-Delgado et al., 2006). In this study, we first determined the transcriptomic features of the left atrium in SHRs. GO, GSEA, and KEGG pathway analysis suggested that the regulated transcripts were attributed to multiple functions, such as TGF-beta signaling pathway, SMAD signaling pathway, fatty acid metabolism, oxidative phosphorylation, the citrate cycle, propanoate metabolism, NF-κB pathway, MAPK, and calcium signaling pathway, which could be associated with atrial fibrosis, inflammation, electrical remodeling, and metabolic changes. According to the KDA analysis of DEGs and further relation network with KEGG pathway analysis, most key driver genes were involved in cardiac metabolism, such as fatty acid metabolism, carbon metabolism, propanoate metabolism and PPAR signaling pathway, suggesting these pathways may play pivotal roles in the pathophysiology of atrial fibrillation in hypertension. Meanwhile, we noticed that *Hadha*, *Hadhb*, and *Eci2*, the top three of the hub genes, were both related to fatty acid metabolism. Cardiac remodeling is characterized by metabolic remodeling, especially down-regulation of fatty acid oxidation, which can further aggravate pathological remodeling (Kolwicz et al., 2013; Mouton et al., 2020). Hadha and Hadhb play a key role in fatty acid oxidation and cardiolipin remodeling, and are involved in cardiac remodeling and systolic dysfunction in heart failure (Le et al., 2014; Miklas et al., 2019; Dagher et al., 2021). The expression of PPAR and medium chain Acyl CoA dehydrogenase was decreased in 4-month-old SHRs (Purushothaman et al., 2011). PPAR activation and increased fatty acid metabolism were observed in SHRs after 4 months treatment of medium-chain triglycerides, accompanied by reduction of oxidative stress and improvement of myocardial hypertrophy (Saifudeen et al., 2017). Our results proved that genes involved in fatty acid metabolism were significantly dysregulated before the onset of heart failure, even before cardiac structural changes, suggesting that fatty acid metabolim may be involved in the structural remodeling of left atrium at the early stage of hypertension.

The following contents will describe the transcriptional characteristics of hypertensive left atrial in terms of atrial fibrosis, cardiac metabolism, cardiac inflammation, and electrical remodeling.

#### 4.1.1 Fibroblast proliferation and atrial fibrosis

In the present study, genes involved in the TGF-β signaling pathway were significantly dysregulated. The TGF-β1 pathway is involved in the development and propagation of AF. The TGF-β1 pathway is linked to atrial fibrosis, and the most common mechanisms involved include the SMAD signaling pathway, the endothelial to mesenchymal transition, and the CD44 signaling pathway (Babapoor-Farrokhran et al., 2021). A recent study showed that serum levels of TGF-β1 gradually increased in the following four groups: control group, hypertensive patients, paroxysmal AF secondary to hypertension, and chronic AF secondary to hypertension, thereby demonstrating that TGF-β1 may contribute to the initiation and sustainment of AF in hypertensive patients *via* atrial remodeling and fibrosis (Lin et al., 2015). The upregulated expression of TGF-β in the atrium results in increased collagen I and III fibrosis, and pirfenidone significantly reduces arrhythmogenic atrial remodeling by suppressing TGF-β1 expression (Lee et al., 2006; Kong et al., 2014). In summary, hypertension may lead to left atrial fibrosis and structural remodeling, and further increase the susceptibility to AF by upregulating TGF-β1.

#### 4.1.2 Cardiac metabolic remodeling

The abnormal metabolic milieu is considered a critical amplifier in cardiac injury during hypertension and it plays an essential role in AF (Pfeffer et al., 2019). In a previous study of the early stage of hypertension development, profound changes in metabolites were observed before the impairment of cardiac function, which comprised increased glucose uptake and oxidation, an increased substrate supply, and elevated pyruvate and fatty acyl groups (Li et al., 2019). Abnormal myocardial fatty acid metabolism was shown to induce the incidence and persistence of AF (Shingu et al., 2020). Changes in fatty acid metabolism, oxidative phosphorylation, and the citrate cycle were also observed in our study. Mitochondrial dysfunction is a significant feature of the heart in hypertensive patients and it leads to the transformation of metabolism to glycolysis (Zhang et al., 2015). Nevertheless, insulin resistance reduces the utilization of glucose, which further aggravates myocardial injury (Mouton et al., 2020). Similarly, in our study, we observed significant changes in genes associated with mitochondria and insulin resistance.

#### 4.1.3 Oxidative stress and inflammation

Both NF-κB and MAPK can be activated by toll-like receptors to increase the expression of cytokines such as IL-6 and TNF, which induce inflammation, which participates in atrial remodeling (Kawano et al., 2005; Kawai and Akira, 2010). NF-κB may be involved in the oxidative stress process through the phosphatidylinositol 3-kinase/protein kinase B pathway, which is a common signal that cross-links with nuclear factor E2-related factor 2 (Nrf2) (Jayasooriya et al., 2014). Inhibition of NF-κB has been shown to activate Nrf2, which protects the cardiovascular system from pathological cardiac remodeling by reducing oxidative stress responses (Zhou et al., 2014). We found that genes associated with the NF-κB pathway were significantly dysregulated in the left atrium of SHRs, thereby suggesting that overloaded hypertension may induce atrial remodeling through NF-κB and further increase the incidence of AF. Hypertension-induced atrial remodeling activates hypoxia-inducible factor-1(HIF-1), which further activates monocyte libraries and proinflammatory cytokines (Rius et al., 2008; Fujisaka et al., 2013).

#### 4.1.4 Ion channel, cell junction, and electrical remodeling

High hydrostatic pressure has been shown to affect the expression of potassium and calcium channels in the left auricle in SHRs and lead to electrical remodeling of the left atrium (Li et al., 2020). Similarly, we found that genes related to ion channels were significantly dysregulated in the left atrium of SHRs. The mechanisms associated with AF include triggers that generate ectopic activity or modifiers of substrate promoted re-entry (Thomas and Abhayaratna, 2017). Electrical remodeling plays a crucial role in AF and its molecular mechanism is based on ion channel expression and/or phosphorylation (Schotten et al., 2011). In particular, electrical reconstruction promoted ion channel (decreased L-type Ca2+ current, rectifier background K+ current) changes to result in a shortened atrial effective refractory period, prolonged excitability interval, and facilitated re-entry (Wiedmann et al., 2018; Dridi et al., 2020).

### 4.2 Potential mechanisms of reversing atrial remodeling by ARB/ARNI

In the present study, we observed a common metabolism-related gene change in the ARB-treated and ARNI-treated groups. Renin–angiotensin system blockers can potentially improve cardiometabolic parameters, such as insulin resistance, glucose metabolism, and adipose tissue dysfunction (Jahandideh and Wu, 2020). Interestingly, these regulated pathways involved in cardiac metabolism, especially regulated by ARNI, such as fatty acid elongation and propanoate metabolism, were also dysregulated in the SHR. Therefore, we speculate that ARNI and ARB may reverse atrial remodeling by uniformly alleviating cardiometabolic dysfunction. Previous studies have shown that ARNI can improve cardiac function in patients with heart failure by improving ventricular fibrosis, reducing cardiac hypertrophy and cardiac inflammation (Lara et al., 2012; Pascual-Figal et al., 2021), while cardiac metabolism was rarely mentioned. The main source of energy consumed by healthy myocardium is fatty acid oxidation, whereas a shift from free fatty acid to glucose utilization is observed in failing heart (Li et al., 2019). Our results provide a new insight for the application of ARNI in the early prevention of heart failure caused by overloaded pressure.

Given the superior prognosis when treating cardiovascular disease with sacubitril/valsartan compared with ARB, we compared the differences in the left atrium under treatment with these drugs. The results showed that the regulated mRNAs were enriched in ECM-receptor interactions and the cGMP-PKG signaling pathway. Notably, genes involved in cGMP-PKG signaling pathway were up-regulated in SHR, but the expression changes of these genes were reversed in the sacubitril/valsartan treated rats. As an inhibitor of endopeptidase enzyme neprilysin, sacubitril/valsartan reduces natriuretic peptides (NPs) degradation and lead to enhanced NP action (Ishii et al., 2017). NPs act as key negative regulators during cardiac hypertrophy and remodeling by activating cGMP-dependent PKG (Takimoto, 2012; Kong and Blanton, 2013). A recent study has shown that sacubitril/valsartan can significantly improve stress-induced myocardial fibrosis by regulating atrial natriuretic peptide-induced PKG signaling in cardiac fibroblasts and inhibiting the expression of fibroblast transformation-related processes, which are not generated by treatment with the molar equivalent of valsartan (Burke et al., 2019), and our results are consistent with these changes. Besides, sacubitril valsartan was shown to significantly increase circulating cGMP levels in beagles compared with valsartan (Mochel et al., 2019). Therefore, we hypothesized that sacubitril/valsartan may reverse hypertension-induced left atrial remodeling through cGMP-PKG signaling pathway, which need further *in vivo* and *in vitro* experiments to confirm.

## 5 Conclusion

In this study, we employed transcriptomic analysis using RNA-seq to determine the changes in the gene expression levels in the left atrium in SHRs compared with WKY rats, and SHRs under treatment with anti-hypertension drugs. Intensive bioinformatics analysis identified atrial fibrosis, inflammation, electrical remodeling, and metabolism changes as critical BPs, and essential pathways were also identified under sacubitril/valsartan and ARB interventions. Meanwhile, we emphasize the importance of cardiac metabolic remodeling and Rac1 in inducing and reversing left atrial remodeling at the early stage of hypertension. Overall, the results obtained in this study might provide insights into the underlying mechanisms associated with the AF substrate in spontaneous hypertension and potential treatment targets for preventing the incidence of AF in hypertension.

## Data Availability

The datasets presented in this study can be found in online repositories. The names of the repository/repositories and accession number(s) can be found below: https://www.ncbi.nlm.nih.gov; GSE207283.
