# Supplementary material for: Transcriptomic profile analysis of the left atrium in spontaneously hypertensive rats in the early stage
Source: Front Pharmacol. 2022 Oct 17;13:989636. doi: 10.3389/fphar.2022.989636 (PMC9620422; doi:10.3389/fphar.2022.989636)
Supplement: Supplementary file 1 [file DataSheet1.docx]

Supplementary Material

# Supplementary Data

All RNA- sequencing data have been uploaded to the GEO database.To review GEO accession GSE207283: Go to https://www.ncbi.nlm.nih.gov/geo/query/acc.cgi?acc=GSE207283. Enter token ihmjsaoahhorzsb into the box.

All the image data of left atrial tissue sections, including HE, Masson and IHC, have been uploaded to nut Cloud. (https://www.jianguoyun.com/c/sd/157fd3a/6ba82530c228f556)

# Supplementary Figures and Tables

## Supplementary Figures


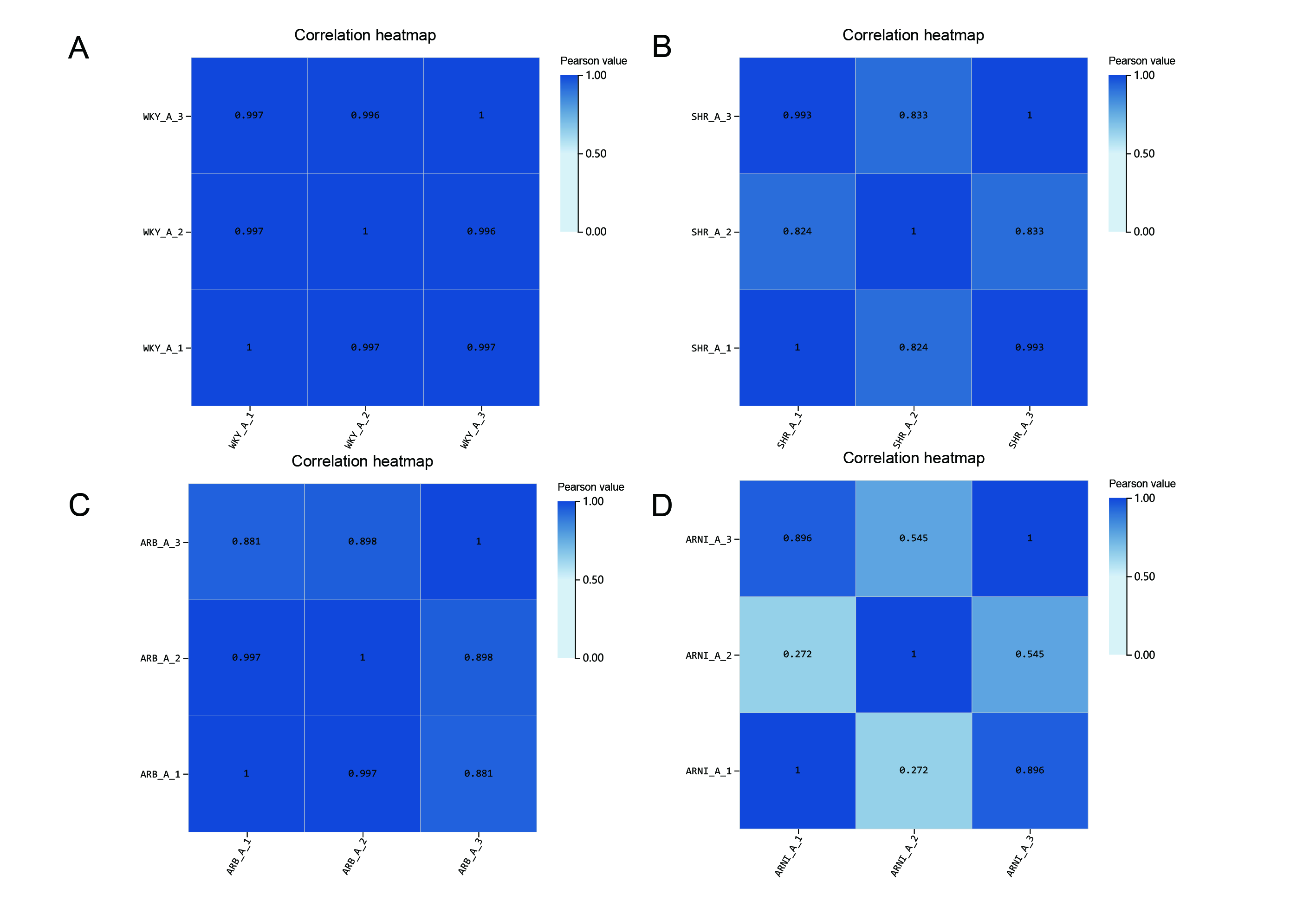


**Supplementary Figure 1.** correlation heatmap analysis of WKY rats (A), SHR (B), ARB-treated group (C), and ARNI-treated group (D).

## Supplementary Tables

| ratio of inflammatory cell infiltration and necrosis area to the entire field | score |
| --- | --- |
| none | 0 |
| 0-25% | 1 |
| 25%-50% | 2 |
| 50%-75% | 3 |
| 75%-100% | 4 |

**Supplementary Table 1.** Scoring criteria for HE staining sections.

| sections | necrosis score | inflammatory score |
| --- | --- | --- |
|  |  |  |
| ARB1 1 200-1 | 0 | 0 |
| ARB1 1 200-2 | 0 | 0 |
| ARB1 1 200-3 | 0 | 0 |
| ARB1 2 200-1 | 0 | 0 |
| ARB1 2 200-2 | 0 | 0 |
| ARB1 2 200-3 | 0 | 0 |
| ARB1 3 200-1 | 0 | 0 |
| ARB1 3 200-2 | 0 | 0 |
| ARB1 3 200-3 | 0 | 0 |
| ARB1 7 200-1 | 0 | 0 |
| ARB1 7 200-2 | 0 | 0 |
| ARB1 7 200-3 | 0 | 0 |
| ARNI1 1 200-1 | 0 | 0 |
| ARNI1 1 200-2 | 0 | 0 |
| ARNI1 1 200-4 | 0 | 0 |
| ARNI1 2 200-1 | 0 | 0 |
| ARNI1 2 200-2 | 0 | 0 |
| ARNI1 2 200-5 | 0 | 1 |
| ARNI1 3 200-1 | 0 | 0 |
| ARNI1 3 200-2 | 0 | 0 |
| ARNI1 3 200-3 | 0 | 1 |
| ARNI1 7 200-1 | 0 | 0 |
| ARNI1 7 200-2 | 0 | 0 |
| ARNI1 7 200-4 | 0 | 0 |
| SHR1 1 200-3 | 0 | 0 |
| SHR1 1 200-4 | 0 | 0 |
| SHR1 1 200-5 | 0 | 0 |
| SHR1 2 200-2 | 0 | 0 |
| SHR1 2 200-3 | 0 | 0 |
| SHR1 2 200-4 | 0 | 0 |
| SHR1 3 200-2 | 0 | 0 |
| SHR1 3 200-3 | 0 | 0 |
| SHR1 3 200-4 | 0 | 0 |
| SHR1 7 200-3 | 0 | 0 |
| SHR1 7 200-4 | 0 | 0 |
| SHR1 7 200-5 | 0 | 0 |
| WKY1 1 200-1 | 0 | 0 |
| WKY1 1 200-2 | 0 | 0 |
| WKY1 1 200-3 | 0 | 0 |
| WKY1 2 200-1 | 0 | 0 |
| WKY1 2 200-2 | 0 | 0 |
| WKY1 2 200-3 | 0 | 0 |
| WKY1 3 200-1 | 0 | 0 |
| WKY1 3 200-2 | 0 | 0 |
| WKY1 3 200-3 | 0 | 0 |
| WKY1 7 200-1 | 0 | 0 |
| WKY1 7 200-2 | 0 | 0 |
| WKY1 7 200-3 | 0 | 0 |

**Supplementary Table 2.** Scores of different HE section fields in the 4 groups.

| Name | IOD | Area | AREAL DENSITY |
| --- | --- | --- | --- |
| ARB1 3 masson 200-1 | 11998.79 | 722352 | 0.016611 |
| ARB1 3 masson 200-2 | 11130.45 | 1121625 | 0.009924 |
| ARB1 3 masson 200-3 | 11977.4 | 1262580 | 0.009486 |
| ARB1 8 masson 200-1 | 3562.262 | 1128003 | 0.003158 |
| ARB1 8 masson 200-2 | 2816.074 | 913540 | 0.003083 |
| ARB1 8 masson 200-3 | 755.1847 | 1168635 | 0.000646 |
| ARB1 2 masson 200-1 | 16025.62 | 822071 | 0.019494 |
| ARB1 2 masson 200-2 | 7427.617 | 967129 | 0.00768 |
| ARB1 2 masson 200-3 | 16409.02 | 688202 | 0.023843 |
| ARB1 4 masson 200-1 | 7852.616 | 671024 | 0.011702 |
| ARB1 4 masson 200-2 | 22740.27 | 658161 | 0.034551 |
| ARB1 4 masson 200-3 | 10507 | 693930 | 0.015141 |
| ARNI 1 3 masson 200-1 | 25005.4 | 1006685 | 0.024839 |
| ARNI 1 3 masson 200-2 | 31104.92 | 1466486 | 0.021211 |
| ARNI 1 3 masson 200-3 | 11710.19 | 1260859 | 0.009287 |
| ARNI 1 8 masson 200-1 | 7095.323 | 1265609 | 0.005606 |
| ARNI 1 8 masson 200-2 | 8014.805 | 1442062 | 0.005558 |
| ARNI 1 8 masson 200-3 | 7412.578 | 1436891 | 0.005159 |
| ARN1 1 2 masson 200-1 | 1168.784 | 1101163 | 0.001061 |
| ARN1 1 2 masson 200-2 | 17955.32 | 731467 | 0.024547 |
| ARN1 1 2 masson 200-3 | 10924.21 | 836987 | 0.013052 |
| ARN1 1 4 masson 200-1 | 10278.23 | 709737 | 0.014482 |
| ARN1 1 4 masson 200-2 | 15173.25 | 834899 | 0.018174 |
| ARN1 1 4 masson 200-3 | 6249.282 | 832555 | 0.007506 |
| SHR 1 3 masson 200-1 | 9669.349 | 855598 | 0.011301 |
| SHR 1 3 masson 200-2 | 12869.23 | 1001946 | 0.012844 |
| SHR 1 3 masson 200-3 | 2139.391 | 1336908 | 0.0016 |
| SHR 1 8 masson 200-1 | 7561.247 | 830800 | 0.009101 |
| SHR 1 8 masson 200-2 | 8164.092 | 1133536 | 0.007202 |
| SHR 1 8 masson 200-3 | 8100.14 | 913145 | 0.008871 |
| SHR1 2 masson 200-1 | 6694.145 | 805752 | 0.008308 |
| SHR1 2 masson 200-2 | 5860.371 | 880386 | 0.006657 |
| SHR1 2 masson 200-3 | 5307.847 | 815933 | 0.006505 |
| SHR1 4 masson 200-1 | 10428.91 | 955894 | 0.01091 |
| SHR1 4 masson 200-2 | 13083.93 | 850332 | 0.015387 |
| SHR1 4 masson 200-3 | 6224.419 | 756392 | 0.008229 |
| WKY 1 3 masson 200-1 | 3207.638 | 1388478 | 0.00231 |
| WKY 1 3 masson 200-2 | 3295.61 | 1371174 | 0.002403 |
| WKY 1 3 masson 200-3 | 1868.105 | 1474560 | 0.001267 |
| WKY 1 8 masson 200-1 | 5303.481 | 914094 | 0.005802 |
| WKY 1 8 masson 200-2 | 5157.273 | 964917 | 0.005345 |
| WKY 1 8 masson 200-3 | 1266.487 | 1266750 | 0.001 |
| WKY 1 2 masson 200-1 | 6326.89 | 1187714 | 0.005327 |
| WKY 1 2 masson 200-2 | 2494.432 | 1328619 | 0.001877 |
| WKY 1 2 masson 200-3 | 280.3143 | 1382025 | 0.000203 |
| WKY 1 4 masson 200-1 | 129.3317 | 1368723 | 0.000094 |
| WKY 1 4 masson 200-2 | 528.7747 | 1060642 | 0.000499 |
| WKY 1 4 masson 200-3 | 36.49689 | 1444924 | 0.000025 |

**Supplementary Table 3.** Areal density of different Masson section field in the 4 groups.

| Name | area of collagen fibers | Area | area ratio of collagen fibers（%） |
| --- | --- | --- | --- |
| ARB1 2 masson 200-1 | 71275 | 722352 | 9.867073 |
| ARB1 2 masson 200-2 | 73582 | 1121625 | 6.560303 |
| ARB1 2 masson 200-3 | 79331 | 1262580 | 6.283245 |
| ARB1 8 masson 200-1 | 26228 | 1128003 | 2.325171 |
| ARB1 8 masson 200-2 | 18323 | 913540 | 2.005714 |
| ARB1 8 masson 200-3 | 4908 | 1168635 | 0.419977 |
| ARB1 3 masson 200-1 | 74280 | 822071 | 9.0357159 |
| ARB1 3 masson 200-2 | 37160 | 967129 | 3.8423003 |
| ARB1 3 masson 200-3 | 83883 | 688202 | 12.188718 |
| ARB1 4 masson 200-1 | 33429 | 671024 | 4.981789 |
| ARB1 4 masson 200-2 | 93210 | 658161 | 14.162188 |
| ARB1 4 masson 200-3 | 43045 | 693930 | 6.2030752 |
| ARNI 1 2 masson 200-1 | 177677 | 1006685 | 17.64971 |
| ARNI 1 2 masson 200-2 | 199341 | 1466486 | 13.59311 |
| ARNI 1 2 masson 200-3 | 70996 | 1260859 | 5.630764 |
| ARNI 1 8 masson 200-1 | 48414 | 1265609 | 3.825352 |
| ARNI 1 8 masson 200-2 | 52676 | 1442062 | 3.652825 |
| ARNI 1 8 masson 200-3 | 46009 | 1436891 | 3.201983 |
| ARN1 1 3 masson 200-1 | 4730 | 1101163 | 0.4295459 |
| ARN1 1 3 masson 200-2 | 72794 | 731467 | 9.9517818 |
| ARN1 1 3 masson 200-3 | 42657 | 836987 | 5.0964949 |
| ARN1 1 4 masson 200-1 | 41916 | 709737 | 5.9058496 |
| ARN1 1 4 masson 200-2 | 65043 | 834899 | 7.7905232 |
| ARN1 1 4 masson 200-3 | 26872 | 832555 | 3.2276546 |
| SHR 1 2 masson 200-1 | 62223 | 855598 | 7.272457 |
| SHR 1 2 masson 200-2 | 77406 | 1001946 | 7.725566 |
| SHR 1 2 masson 200-3 | 15129 | 1336908 | 1.131641 |
| SHR 1 8 masson 200-1 | 48590 | 830800 | 5.84858 |
| SHR 1 8 masson 200-2 | 53146 | 1133536 | 4.688515 |
| SHR 1 8 masson 200-3 | 56272 | 913145 | 6.162439 |
| SHR 1 3 masson 200-1 | 35850 | 805752 | 4.4492598 |
| SHR 1 3 masson 200-2 | 30503 | 880386 | 3.4647302 |
| SHR 1 3 masson 200-3 | 32976 | 815933 | 4.0415083 |
| SHR 1 4 masson 200-1 | 43213 | 955894 | 4.5206895 |
| SHR 1 4 masson 200-2 | 51630 | 850332 | 6.0717461 |
| SHR 1 4 masson 200-3 | 28231 | 756392 | 3.732324 |
| WKY 1 2 masson 200-1 | 16683 | 1388478 | 1.201531 |
| WKY 1 2 masson 200-2 | 19067 | 1371174 | 1.39056 |
| WKY 1 2 masson 200-3 | 10829 | 1474560 | 0.734389 |
| WKY 1 8 masson 200-1 | 31135 | 914094 | 3.406105 |
| WKY 1 8 masson 200-2 | 26670 | 964917 | 2.763968 |
| WKY 1 8 masson 200-3 | 7514 | 1266750 | 0.593172 |
| WKY 1 3 masson 200-1 | 20921 | 1187714 | 1.761451 |
| WKY 1 3 masson 200-2 | 9258 | 1328619 | 0.6968138 |
| WKY 1 3 masson 200-3 | 1372 | 1382025 | 0.0992746 |
| WKY 1 4 masson 200-1 | 469 | 1368723 | 0.0342655 |
| WKY 1 4 masson 200-2 | 2121 | 1060642 | 0.1999732 |
| WKY 1 4 masson 200-3 | 197 | 1444924 | 0.0136339 |

**Supplementary Table 4.** The area ratio of collagen fibers of different Masson section field in the 4 groups.

| Names of Images | Mean Density |
| --- | --- |
| SHR1 TGF-β 6 200-1 | 0.409 |
| SHR1 TGF-β 6 200-2 | 0.4221 |
| SHR1 TGF-β 6 200-3 | 0.7559 |
| SHR1 TGF-β 6 200-4 | 0.4158 |
| SHR1 TGF-β 6 200-5 | 0.3884 |
| SHR1 TGF-β 6 200-6 | 0.3869 |
| WKY1 TGF-β 3 200-1 | 0.3775 |
| WKY1 TGF-β 3 200-2 | 0.3782 |
| WKY1 TGF-β 3 200-3 | 0.3855 |
| WKY1 TGF-β 3 200-4 | 0.3888 |
| WKY1 TGF-β 3 200-5 | 0.384 |
| WKY1 TGF-β 3 200-6 | 0.3859 |

**Supplementary Table 5.** Mean density of different section in immunohistochemistry analysis in the 4 groups.
